# MAC-sparing effect of nitrous oxide in sevoflurane anesthetized sheep and its reversal with systemic atipamezole administration

**DOI:** 10.1371/journal.pone.0190167

**Published:** 2018-01-09

**Authors:** Lauren Duffee, Nicolò Columbano, Antonio Scanu, Valentino Melosu, Giovanni Mario Careddu, Giovanni Sotgiu, Bernd Driessen

**Affiliations:** 1 Department of Clinical Studies-New Bolton Center, University of Pennsylvania, School of Veterinary Medicine, Kennett Square, Pennsylvania, United States of America; 2 Dipartimento di Medicina Veterinaria, Università degli Studi di Sassari, Sassari, Sardegna, Italy; 3 Centro di Ricerca di Chirurgia Comparata (CRCC), Università degli Studi di Sassari, Sassari, Sardegna, Italy; 4 Dipartimento di Scienze Biomediche, Università degli Studi di Sassari, Sassari, Sardegna, Italy; 5 Narkovet Consulting™ LLC, Chadds Ford, PA, United States of America; University of Bari, ITALY

## Abstract

**Introduction:**

Nitrous oxide (N_2_O) is an anesthetic gas with antinociceptive properties and reduces the minimum alveolar concentration (MAC) for volatile anesthetic agents, potentially through mechanisms involving central alpha_2_-adrenoceptors. We hypothesized that 70% N_2_O in the inspired gas will significantly reduce the MAC of sevoflurane (MAC_SEVO_) in sheep, and that this effect can be reversed by systemic atipamezole.

**Materials and methods:**

Animals were initially anesthetized with SEVO in oxygen (O_2_) and exposed to an electrical current as supramaximal noxious stimulus in order to determine MAC_SEVO_ (in duplicates). Thereafter, 70% N_2_O was added to the inspired gas and the MAC re-determined in the presence of N_2_O (MAC_SN_). A subgroup of sheep were anesthetized a second time with SEVO/N_2_O for re-determination of MAC_SN_, after which atipamezole (0.2 mg kg^-1^, IV) was administered for MAC_SNA_ determinations. Sheep were anesthetized a third time, initially with only SEVO/O_2_ to re-determine MAC_SEVO_, after which atipamezole (0.2 mg kg^-1^, IV) was administered for determination of MAC_SA_.

**Results:**

MAC_SEVO_ was 2.7 (0.3)% [mean (standard deviation)]. Addition of N_2_O resulted in a 37% reduction of MAC_SEVO_ to MAC_SN_ of 1.7 (0.2)% (p <0.0001). Atipamezole reversed this effect, producing a MAC_SNA_ of 3.1 (0.7)%, which did not differ from MAC_SEVO_ (p = 0.12). MAC_SEVO_ did not differ from MAC_SA_ (p = 0.69). Cardiorespiratory variables were not different among experimental groups except a lower ETCO_2_ in animals exposed to SEVO/N_2_O.

**Conclusions:**

N_2_O produces significant MAC_SEVO_-reduction in sheep; this effect is completely reversed by IV atipamezole confirming the involvement of alpha_2_-adrenoreceptors in the MAC-sparing action of N_2_O.

## Introduction

Nitrous oxide (N_2_O) is a commonly used gas anesthetic in human medicine but due to its lower efficacy in animal species [1] is currently sparingly utilized in veterinary anesthesia. Why N_2_O exhibits greater potency in humans than animals remains unknown [1–4]. Currently, the mechanism of action of the anesthetic and analgesic effects of N_2_O remains incompletely defined. With regard to its antinociceptive/analgesic actions, studies suggest an involvement of alpha_2_-adrenoceptors at the spinal level [5–7]. Therefore, incorporation of N_2_O into a balanced anesthetic protocol may help reduce the amount of volatile anesthetic required [8–10], and provide pronounced antinociception.

The current study was performed to determine the impact of N_2_O on the MAC of sevoflurane (SEVO) in anesthetized sheep. We hypothesized that 70% N_2_O, when combined with the volatile agent, will markedly reduce MAC_SEVO_ and that this effect is mediated by activation of alpha_2_-adrenoceptors within the spinal cord and possibly elsewhere in the central nervous system. We further speculated that administration of atipamezole, the currently most selective, competitive alpha_2_-adrenoceptor antagonist, would reverse the MAC-sparing effect.

## Materials and methods

Fourteen systemically healthy, ASA status I, female Sardinian sheep (2–8 years old) were included. The study protocol was approved by the Institutional Animal Care and Use Committee at the University of Sassari (CIBASA; protocol number 22859) according to Italian legislation and was conducted in compliance with the NIH Guide for the Care of Laboratory Animals in its current form and Directive 2010/63/EU revising Directive 86/609/EEC on the protection of animals used for scientific purposes as adopted on 22 September 2010.

### Pre-experimental animal preparation

Food was withheld from all sheep for 12 h prior to anesthesia, but free access to water was allowed until one h before beginning the experimental procedure. Pre-anesthetic body weight (kg), heart rate (HR, min^-1^), respiratory rate (RR, min^-1^), and rectal temperature (T,°C) were recorded. The sheep were positioned in lateral recumbency. The rostral auricular artery was catheterized after the area was clipped, aseptically prepared, and locally infiltrated with subcutaneous lidocaine 2%. The area over the lateral saphenous vein was similarly prepared and the vein catheterized.

No premedication was administered. The sheep were pre-oxygenated for 3 min with oxygen (O_2_) at 3 L min^-1^ via a tight-fitting face mask connected to a standard rebreathing system, which was attached to a workstation (Perseus^®^, Dräger, Lübeck, Germany) that operates with a ventilator rebreathing system (Ventilator TurboVent2^®^, Dräger, Lübeck, Germany) and sodalime (Drägersorb^®^, Dräger, Lübeck, Germany) as CO_2_ absorbent. During this time, baseline measurements included HR, invasive systolic (SAP), mean (MAP), and diastolic (DAP) arterial blood pressures (mmHg), arterial oxygenation (SPO_2,_ %) and the electrocardiogram (ECG). Saline 0.9% was administered at a rate of 5 mL kg^-1^ h^-1^.

Anesthesia was induced with SEVO in O_2_ delivered at a vaporizer dial setting of 8% via a face mask for at least 3 min. When an adequate depth of anesthesia was achieved, determined by a lack of palpebral reflex, decreased jaw tone, and cessation of swallowing, the sheep were intubated with a 9.0-mm (ID) Murphy cuffed endotracheal tube, subsequently connected to the anesthetic circuit. Mechanical ventilation was initiated and re-adjusted to achieve normocapnea (PaCO_2_, 5.3–6.6 kPa). For this purpose, arterial blood was collected in a heparin coated syringe and immediately examined using a cartridge-based blood gas analyzer (ABL 80 CO-OX flex^®^, Radiometer, Copenhagen, Denmark).

The HR, ECG, invasive arterial blood pressures, SpO_2_, and esophageal body temperature (T,°C) were continuously monitored using a multiparameter monitor (Infinity Delta^®^ XL, Dräger, Lübeck, Germany). The RR, tidal volume (VT, mL), fresh gas (O_2_/N_2_O) rate (constantly 3 L min^-1^) were monitored using the anesthesia workstation (Perseus A500^®^, Dräger, Lübeck, Germany) screen. The inspired fraction of O_2_ and N_2_O (FiO_2_, FiN_2_O), end-tidal sevoflurane concentration (ET_SEVO_, %) and end-tidal partial pressure of CO_2_ (ET_CO2_, kPa) were continuously monitored with the integrated multi-gas module SCIO (Dräger, Lübeck, Germany) operating as a side stream analyzer (250 mL min^-1^), which was calibrated prior to each experiment using gas standards (1% SEVO, 5% CO_2_; calibration gas, Air Liquide Healthcare America, Plumsteadville, Pennsylvania, USA).

To deliver the noxious stimulus, disposable low-resistance silver/silver chloride electrodes with an inter-electrode distance of 1 cm (Norotrode 20 Bipolar SEMG Electrodes, Myotronics-Noromed, Inc., WA, USA) were applied on the lateral metacarpal surface midway between the carpus and metacarpophalangeal joints and secured with auto-adhesive wrap (Vetrap TM; St. Paul, Minnesota, USA). The electrodes were connected to a 220-volt powered constant current stimulator, model DS7A (Digitimer Ltd., Letchworth Garden City, UK) that delivered electrical impulses when triggered by a 9-volt battery powered train/delay generator, model DG2A (Digitimer Ltd., Letchworth Garden City, UK). Trains of square-wave impulses (1 ms in duration; 5 Hz, 50 mA constant current) were delivered for 60 s or until a gross purposeful motor response was noted, after which the stimulator was switched off. As the maximum delivered voltage was 200 V, the electrical resistance between electrodes was measured using an ohmmeter (Personal 20; Mega Elettronica, Milan, Italy) prior to each stimulation to ensure resistance remained at < 3 kΩ necessary to discharge a current of 50 mA. If resistance had increased to > 3 kΩ, electrodes were exchanged.

### Determination of MAC_SEVO_ and MAC_SEVO_ with N_2_O (MAC_SN_)

A modified protocol of “up-down” method as proposed by Dixon was employed [11]. After a 30-min equilibration period at an ET_SEVO_ of 2.7%, electrical stimulation was applied, and sheep were examined for gross purposeful movement. A response was positive when major motor activity was observed in non-stimulated body parts, such as flexion or extension of the contralateral hind limb or gross neck movements. Muscle tremors, occasional swallowing, nystagmus and changes in cardio-respiratory parameters did not represent a positive response. Depending on the presence or lack of a positive response, the vaporizer dial setting was increased or decreased, respectively, to arrive at an ET_SEVO_ 0.2% higher or lower than previously recorded. The new ET_SEVO_ concentration was maintained for 15 min before the electrical stimulation was repeated.

The MAC value was calculated as the arithmetic mean of two ET_SEVO_ concentrations: the value measured when the response had been positive, and the subsequent higher value recorded when the response had been negative. Each determination of MAC_SEVO_ was performed in duplicate. After MAC_SEVO_ determination was completed, 70% N_2_O was added to the inspired gas maintaining an overall fresh gas flow rate of 3 L min^-1^. After a 30-min equilibration, the procedure was repeated following the protocol described above, for duplicate MAC_SN_ determination.

### Determination of MAC_SN_ and MAC_SEVO_ with N_2_O in presence of atipamezole (MAC_SNA_)

After a 10-d washout period, six sheep were re-anesthetized using the tight-fitting face mask as described above. However, the anesthesia machine was now set to deliver a total fresh gas flow of 3 L min^-1^ with an inspired concentration of N_2_O of 70% (FiN_2_O = 0.70) in the carrier gas, with the FiO_2_ automatically calculated and adjusted by the anesthesia workstation based on the set FiN_2_O. During induction of anesthesia the SEVO vaporizer dial setting was 8%. The carrier gas mixture including 70% N_2_O was continuously delivered at a rate of 3 L min^-1^ throughout the experimental procedure. After a 30-min equilibration at an ET_SEVO_ corresponding to each animal’s previously determined MAC_SN_, the MAC_SN_ was re-determined in duplicate as described. Subsequently, atipamezole (0.2 mg kg^-1^) was administered IV following the pharmacodynamic and pharmacokinetic profile of this antagonist described in sheep [12]. If the MAC_SNA_ determinations extended past 120 min of anesthesia, an additional atipamezole bolus was administered (0.1 mg kg^-1^) [12]. The ET_SEVO_ concentration was adjusted to a concentration corresponding with the individual’s MAC_SEVO_. Sheep were equilibrated at this ET_SEVO_ concentration for 15 min before duplicate determination of MAC_SNA_.

### Determination of MAC_SEVO_ in the presence of atipamezole (MAC_SA_)

Twelve months after completing the MAC_SNA_ experiments, six of the original 12 sheep in the MAC_SEVO_ group were re-anesthetized to determine the effect of IV atipamezole on MAC_SEVO_. The same protocol as described above for the determination of MAC_SEVO_ was followed. After completion of MAC_SEVO_ re-determination, IV atipamezole was administered (0.2 mg kg^-1^) and re-dosed as described above. MAC_SA_ determination was performed in duplicate.

### Statistical analysis

An ad-hoc electronic dataset (Excel, Microsoft Office) was prepared to collect all data. Values for those variables determined at distinct time points during the experiments are presented in Tables 1 and 2. All MAC determinations were pooled for each animal. All quantitative data were tested for normality using a Shapiro-Wilk test. For normally distributed data, means and standard deviations (SD) are presented to describe variables pre-anesthesia and after exposure to the anesthetic agents. Non-parametric data are reported as median (IQR). Student’s t test for dependent data was employed to detect any statistically significant differences. A two-tailed p-value <0.05 was considered as indicating statistically significant differences between data groups. All computations were performed with STATA version 13 (Stata Corp, College Station, Texas, USA) statistical software.

**Table 1:** First and second determinations of MAC_SEVO_, MAC_SN_, MAC_SNA_, MAC_SA_, and MAC_SEVO_ in the form of pooled data.

Variables	No. of animals	1^st^ value	2^nd^ value	p-value
MAC_SEVO_	12	2.7 (0.3)^§§^	2.7 (0.3)^§§^	0.85
MAC_SN_	10	1.7 (0.3)**	1.7 (0.2)**	0.26
MAC_SNA_	6	3.2 (0.7)^§§^	2.9 (0.8)^§§^	0.14
MAC_SA_	6	2.7 (0.6)^§§^	2.4 (0.5)^§§^	0.12

Presented are the mean (SD) values for first and second MAC determinations in the various experimental groups (see text for more details). The p-value in the far-right column refers to a comparison of first and second determinations of each of the MAC data presented.

Statistically significant difference from MAC_SEVO_

*p<0.05

** p<0.01

statistically significant difference from MAC_SN_

^§^p<0.05

^§§^ p<0.01

statistically significant difference from MAC_SNA_

^**†**^p<0.05

^**††**^ p<0.01.

**Table 2:** End-tidal concentrations of SEVO and CO_2_ and physiological variables recorded during MAC determinations.

Variables	Baseline	Exposure to SEVO only	Exposure to SEVO plus N_2_O	Exposure to SEVO plus N_2_O plus atipamezole	Exposure to SEVO plus atipamezole	p-value*	p-value**	p-value***	p-value****
End-tidal concentration of SEVO (%)	-	2.7 (0.4)	1.8 (0.4)	3.2 (0.7)	2.6 (0.5)	<0.0001	0.12	0.001	0.08
Heart rate (min^-1^)	108.0 (28.2)	97.3 (17.2)	117.7 (16.9)	107.5 (10.2)	117.3 (25.0)	<0.0001	0.02	0.11	0.0007
Systolic arterial pressure (mmHg)	123.0 (8.2)	114.0 (9.9)	124.1 (11.5)	108.3 (19.2)	117.8 (16.3)	0.008	0.49	0.02	0.56
Mean arterial pressure (mmHg)	105.3 (7.0)	94.8 (11.6)	106.3 (10.1)	92.1 (18.4)	99.8 (14.5)	0.003	0.55	0.01	0.30
Diastolic arterial pressure (mmHg)	92.1 (6.9)	84.7 (11.5)	95.0 (9.1)	80.4 (18.3)	88.1 (13.7)	0.007	0.34	0.01	0.49
Temperature (°C)	39.4 (0.6)	38.6 (0.8)	38.5 (0.8)	37.8 (1.3)	38.1 (0.5)	0.53	0.45	0.38	0.02
End-tidal CO_2_ (kPa)	-	5.26 (0.44)	4.84 (0.64)	4.97 (0.99)	5.11 (0.36)	0.05	0.17	0.74	0.67
SPO_2_ (%)	-	99.3 (1.0)	92.1 (2.9)	92.7 (4.4)	98.9 (1.3)	<0.0001	0.003	0.84	0.29

Data represent the mean (SD) of values recorded at the time of each MAC determination, when no responses to supramaximal stimulation were recognized and average ET_SEVO_ was at the level indicated in the first row.

*: Comparison between mean values measured during exposure to SEVO only versus exposure to SEVO plus N_2_O.

**: Comparison between mean values measured during exposure to SEVO only versus exposure to SEVO plus N_2_O plus atipamezole.

***: Comparison between mean values during exposure to SEVO plus N_2_O versus exposure to SEVO plus N_2_O plus atipamezole.

****: Comparison between mean values during exposure to SEVO only versus exposure to SEVO plus atipamezole.

## Results

The mean (SD) age of the 14 sheep included in the MAC_SEVO_, MAC_SN_, and MAC_SNA_ groups was 5.9 (2.0) years and 7.0 (2.6) years in the six sheep in the MAC_SA_ group (*p* = 0.39). The mean weight of the sheep was 39.4 (4.4) kg and 41.5 (4.3) kg, at each time point, respectively (*p* = 0.22). Intubation required a median of 1 (1,2) attempt after 6.9 (4.0) min of SEVO inhalation.

MAC_SEVO_ was determined in 12 of 14 sheep. One sheep was eliminated because an abnormal larynx anatomy prevented a regular intubation technique. A second sheep was eliminated before MAC_SEVO_ determination due to a vigorous response to the first electrical stimulation that required an IV rescue anesthetic to avoid harm to the animal. The MAC_SN_ was determined in 10 of 12 sheep of the MAC_SEVO_ group. Two sheep demonstrated, in the presence of N_2_O, persistent gross motor activity in response to noxious stimulation that did not, as usual, cease shortly after discontinuing stimulation but persisted for >45 min despite increasing ET_SEVO_ to >5.0%. The MAC_SNA_ was measured in eight sheep, although only successfully determined in six sheep. Two sheep were removed from the MAC_SNA_ determination experiments due to a similar onset of persistent motor activity after noxious stimulation, either before or after atipamezole administration.

Repetitive electrical stimulation produced no tissue injury.

All MAC determinations are summarized in Table 1. The MAC_SEVO_ in the present animal group was 2.7 (0.3) %, while the mean MAC_SN_ was 1.7 (0.3) %, approximately 37% lower than MAC_SEVO_ (p <0.0001). After IV administration of atipamezole, the MAC_SNA_ was 3.1 (0.7) %, which was not statistically different from MAC_SEVO_ (p = 0.12). The negative control group, MAC_SA_, did not differ from MAC_SEVO_ (p = 0.69). As the statistical analysis of first and second MAC values in each study group (see Table 1) revealed, the first and second MAC determinations did not differ significantly from each other. Furthermore, MAC_SEVO_ determinations obtained in the same sheep (Group MAC_SA_) one year later were not different from originally obtained values (p = 0.55), justifying inclusion of those data into the MAC_SEVO_ data pool.

Table 2 lists the ET_SEVO_ and ETCO_2_ and physiological variables recorded in all study groups. When compared to anesthesia with SEVO alone, SEVO in combination with N_2_O and atipamezole, or SEVO in combination with atipamezole, exposure to SEVO/N_2_O produced a significant increase in HR, SAP, MAP, and DAP. The ETCO_2_ decreased significantly during SEVO/N_2_O exposure. The SPO_2_ during SEVO/N_2_O anesthesia was significantly lower than SEVO/O_2_ experiments, but did not produce clinically relevant hypoxemia. Other variables did not differ among any of the study groups.

The MAC_SEVO_ and MAC_SN_, MAC_SNA_, and MAC_SA_ determinations were completed within 405 (106), 387 (42), and 342 (62) min, respectively. The N_2_O exposure in the MAC_SN_ and MAC_SNA_ groups lasted 210 (85) and 387 (42) min, respectively. All sheep recovered uneventfully from anesthesia. No erythema or swelling of the electrical stimulation sites were observed. The sheep were extubated within 8.4 (5.1) min and stood within 28.6 (22.4) min after discontinuing SEVO administration.

## Discussion

Results of the present study support our key hypotheses that N_2_O exhibits a significant MAC_SEVO_-sparing effect in sheep and that this effect involves activation of alpha_2_-adrenoceptors within the central nervous system and can be reversed by administration of the selective alpha_2_-adrenoceptor antagonist atipamezole. The MAC_SEVO_ in this sheep population was 2.7%. The mean MAC_SEVO_ value determined in present experiments is about 40% higher than the one previously reported in three non-pregnant sheep, 1.92 (0.17%) [13], and about 18% lower than the value previously reported in six other sheep, 3.3% [14]. Such variability in MAC values is common and has been observed for various volatile agents including SEVO in many animal species [15]. Biological variation and methodological differences may account for such a discrepancy. Our MAC_SEVO_ data also compare favorably with data in goats, 2.33 (0.15) % [16].

This is the first study in sheep demonstrating a marked, i.e. 37%, reduction of MAC_SEVO_ by 70% N_2_O added to the inhalant gas mixture. This finding is consistent with prior studies examining the MAC-sparing effect of N_2_O in other animal models and for different volatile anesthetic agents. In dogs, addition of 70% N_2_O decreased the MAC of isoflurane by 32% [10] and of desflurane by 16% [17]; and Steffey and co-workers [1] reported with 70% N_2_O an approximately 34, 31, and 37% MAC-reduction of halothane in dogs, cats, and stump-tail monkeys, respectively. The commonly reported 30–40% MAC-sparing effect of 60–75% N_2_O in animal species might be considered a modest reduction compared to the up to 75% decrease in MAC of volatile agents in human patients [8,9,18]. Yet, it does compare favorably to the MAC-sparing effects of other agents, such as lidocaine, various opioids, or (dex-)medetomidine commonly used as adjuncts in balanced anesthesia regimens in clinical veterinary practice [19]. Insofar N_2_O deserves consideration in both veterinary clinical practice and the laboratory animal setting. This applies particularly when the clinical condition of an animal requires limited dosing of other MAC-sparing analgesic agents and/or does not allow for increasing ET concentrations of a volatile agent. Also in moments of severe noxious stimulation during surgery, N_2_O may provide fast-onset/fast-offset antinociception thanks to its favorable pharmacokinetic properties with minimal compromise in cardiorespiratory stability.

Currently the mechanisms of action of N_2_O as an anesthetic remain incompletely characterized. However, it is a long-standing theory that N_2_O’s anesthetic action is primarily mediated through non-competitive inhibition of the NMDA subtype of glutamate receptors in the brain, although opening of potassium channels with subsequent hyperpolarization of cerebral neurons and decreased neuronal firing may also be involved [20–21]. While still undetermined to what extent those molecular mechanisms contribute to N_2_O’s MAC-sparing effects, our finding of a complete reversal of the MAC_SEVO_-sparing effect of N_2_O by IV administration of atipamezole supports the idea that much, if not all, of N_2_O’s MAC-reducing effect is the result of enhanced alpha_2_-adrenoreceptor activity, and therefore potentially from antinociceptive actions. Those complex mechanisms were summarized in a comprehensive review [21], according to which, N_2_O promotes the release of corticotrophin-releasing factor (CRF) from the hypothalamus, which results in increased release of endogenous opioid peptides in the periaqueductal grey in the midbrain. These opioid peptides inhibit the activity of GABA-ergic interneurons in the pons. As a result, descending noradrenergic pathways become more active and release more noradrenaline from their terminals in the spinal cord dorsal horn. There, noradrenaline stimulates alpha_1_-adrenoceptors on GABA-ergic interneurons and alpha_2_-receptors located post-synaptically on the second-order nociceptive neurons. This eventually leads to inhibition of nociceptive impulse conduction to supraspinal centers and thus reduced nociception/pain perception. Insofar, N_2_O seems to act similarly to alpha_2_-agonists such as (dex-) medetomidine and clonidine in the spinal cord, and therefore it may not be surprising that atipamezole could completely reverse its MAC-sparing action.

The determination of MAC_SA_, which did not significantly differ from MAC_SEVO_, shown in Table 1, indicates that atipamezole exhibits no MAC_SEVO_ altering action and that descending noradrenergic pathways are not tonically active and thus do not constantly suppress afferent nociceptive impulse traffic. Our observation coincides with results obtained in previous studies in dogs [22–23]. Consequently, atipamezole seems to only reverse alpha-receptor-dependent antinociceptive/analgesic mechanisms elicited by N_2_O within the spinal cord. A rat study appears to further support this view because only intrathecal, but not intracerebroventricular, atipamezole administration reversed any antinociceptive effect of N_2_O [5].

The majority of sheep in this study showed the typical increased motor activity with noxious stimulation that ceased within min following stimulation and with increase in ET_SEVO_ concentration. However, a puzzling finding in current experiments was that four of the 14 sheep enrolled (29%) demonstrated signs of long-lasting central nervous excitement in response to supramaximal noxious stimulation under SEVO/N_2_O, which required their removal from the study group. The observed excitatory phenomena included gross paddling of multiple limbs, rapid nystagmus, regurgitation, and fasciculation of the neck and leg musculature that persisted for >45 min and could not be stopped by increasing ET_SEVO_ to >5%. Undoubtedly, the noxious stimulus triggered such excitatory phenomena but no pattern was detectable that allowed prediction of such a response. Three sheep responded in such a manner to the first or second noxious stimulation under SEVO/N_2_O anesthesia, despite not displaying excitement during preceding periods of noxious stimulation under SEVO/O_2_ anesthesia. One animal showed excitatory reactions to electrical stimulation only after the administration of atipamezole during an episode of SEVO/N_2_O anesthesia, while not having shown central excitement in a preceding anesthetic under SEVO/N_2_O. Despite demonstrating exaggerated excitation, all four sheep recovered uneventfully, as the other sheep. Since the phenomenon of central agitation post-noxious stimulation was only noticeable in sheep breathing N_2_O, one may speculate whether inadequate suppression of cerebral neuronal activity promoted an unusually strong response of certain brain centers to impulses that arrived at supraspinal sites. While N_2_O might exhibit strong antinociceptive activity at the spinal cord level that is alpha_2_-adrenoceptor mediated, its depressant (i.e. hypnotic/anesthetic) effect at the cerebral level might be weaker, non-uniform, or different in sheep than in other species. Unfortunately, MACN_2_O data have not been reported in sheep but values in other animal species and the human reveal significant inter-species differences. For example, for dogs, cats, pigs, calves, and humans MACN_2_O values of average 236, 255, 211, 223, and 104%, respectively are reported [15].

Interestingly, electroencephalography (EEG) studies in humans indicate that N_2_O can significantly diminish the depressant effects of volatile anesthetics such as isoflurane [24] and sevoflurane [25] on certain cerebral neuronal activity, thereby acting centrally in opposition to the volatile anesthetics. In SEVO-anesthetized human patients, addition of 60% N_2_O produced an 11% increase in the ED_50_ of SEVO for induction of an isoelectric EEG (p < 0.0001), while it did not significantly affect the ED_50_ for electroencephalographic burst suppression [25]. It will require a detailed EEG analysis to potentially elucidate the mechanisms behind the central arousal phenomenon and motor activities observed in some of the SEVO/N_2_O anesthetized sheep in this study.

A limitation for the interpretation of present pulse oximeter data is that no arterial blood gas samples were collected throughout the different phases of the study to more objectively assess the arterial oxygenation status in each animal, particularly when breathing a gas mixture containing 70% N_2_O. In preceding pilot experiments, arterial blood gases had been measured and during the exposure of 70% N_2_O, the lowest PaO_2_ determined was 68 mmHg, which is associated with an arterial oxygen saturation of >90% and thus within a clinically acceptable range given the reduced FiO_2_. We recorded only during the second MAC_SNA_ determination in two sheep temporarily an SpO_2_ of <90% (minimum 83%). Without corresponding arterial blood gas data available, it is difficult to predict the accuracy of those pulse oximeter readings. Nonetheless, those readings do emphasize the need for repetitive arterial blood gas analyses when gas mixtures relatively low in FiO_2_ are used in clinical practice and to increase the FiO_2_ in case arterial oxygen saturation decreases below normal to avoid persistent hypoxemia.

In conclusion, this study demonstrates that N_2_O exerts a significant MAC-reducing effect in sheep. Systemic administration of atipamezole reverses the MAC reduction, supporting the notion that alpha_2_-adrenoceptor dependent mechanisms in the spinal cord dorsal horn and possibly also in the brain are involved in this activity.
